# Editorial: Treat-to-target in systemic lupus erythematosus: cytokine transduction pathways in SLE

**DOI:** 10.3389/fimmu.2024.1503776

**Published:** 2024-10-16

**Authors:** Yuji Nozaki, Yanjie Hao, Jennifer L. Barnas

**Affiliations:** ^1^ Department of Hematology and Collagen Diseases, Kinki University School of Medicine, Osaka, Japan; ^2^ Department of Internal Medicine, St. Vincent's Hospital Melbourne, University of Melbourne, Melbourne, VIC, Australia; ^3^ Department of Rheumatology, St. Vincent's Hospital Melbourne, Melbourne, VIC, Australia; ^4^ Department of Allergy, Immunology and Rheumatology, University of Rochester School of Medicine, Rochester, NY, United States

**Keywords:** cytokine network, SLE, treat to target, novel therapeutic agent, cytokine signaling pathways

Systemic lupus erythematosus (SLE) is a chronic autoimmune disease characterized by dysfunction of T cells, B cells, and antigen-presenting cells. Various cytokine network abnormalities have been reported in both SLE patients and lupus model mice. Some of these abnormalities play important physiological roles in the pathogenesis of the disease, while others are considered bystanders. For example, B-cell activators regulate B-cell maturation and survival, and their imbalance is associated with systemic autoimmunity in SLE and lupus nephritis. Recently, type I interferons (IFNs), particularly IFNα and IFNβ, and their signalling pathways have been implicated in the pathogenesis of SLE, and these cytokines play an important role in lupus. Although these cytokines are thought to be involved in the various pathogenesis of SLE, their exact mechanisms remain unclear.

This Research Topic summarizes six outstanding studies that have been selected by the editors and published. These reports provide a basis for active discussion and publication of research findings by researchers worldwide on cytokine signalling pathways and novel therapeutic strategies in the pathophysiology of SLE. We hope that these studies will contribute to the advancement of SLE research, improve our understanding of disease mechanisms, and improve outcomes for patients.

Tsai et al. reviewed the pathogenesis and potential therapeutic roles of regulatory cell subsets in systemic lupus erythematosus. Regulatory cells play a critical role in maintaining self-tolerance and regulating autoimmunity by dampening self-reactive immune responses. Therefore, an imbalance in regulatory cells can lead to autoimmune and autoinflammatory responses. The authors focused on the function and diversity of specific subsets, including CD4^+^ regulatory T cells, CD8^+^ regulatory T cells, type 1 regulatory T cells, and regulatory B cells. Additionally, they highlighted clinical trials and the therapeutic potential of regulatory cell-based therapies, such as low-dose IL-2 therapy, which aims to restore immune tolerance by modulating Tregs in patients with SLE.

Lv et al. demonstrated that basement membrane (BM)-gene expression in SLE patients and identified six key genes (AGRN, PHF13, SPOCK2, TGFBI, COL4A3, COLQ) for constructing a diagnostic model. Analysis showed AGRN’s significant role in immune functions and its varied expression across different cancers. AGRN’s expression correlated with patient prognosis, immune cell infiltration, and other factors, highlighting its potential importance in both SLE and cancer progression.

Lindblom et al. analysed cytokine and autoantibody levels in SLE patients, healthy controls, and those with other autoimmune diseases using samples from the European PRECISESADS project. Of 83 cytokines tested, 29 were significantly elevated in active SLE compared to healthy controls and other autoimmune diseases, with CCL8, CXCL13, and IL-1RA specifically correlating with higher SLE Disease Activity Index 2000 (SLEDAI-2K) scores. Despite similarities in autoantibody occurrence across SLEDAI-2K organ domains, correlations with disease activity were weak, suggesting that CCL8, CXCL13, and IL-1RA could be promising serum biomarkers for assessing SLE activity upon further validation.

Juvenile systemic lupus erythematosus (jSLE) is a complex autoimmune disorder with significant genetic and transcriptional abnormalities, particularly in the type I interferon (IFN) signaling pathway. Recent studies have highlighted the involvement of IFN signaling components such as JAK1, TYK2, STAT1, and STAT2. In a case report by Rossano et al., the successful treatment of a 13-year-old girl with jSLE, who carried a novel heterozygous missense variant in the TREX1 gene, is detailed. The patient was treated with baricitinib combined with mofetil mycophenolate. TREX1 is crucial for DNA repair, and its mutation is linked to excessive type I IFN production. This case underscores the potential of targeting IFN pathways and highlights the importance of translational research in optimizing treatments for rare diseases like jSLE.

Fan et al. demonstrated, in real-world settings, achieving early lupus low disease activity state (LLDAS) in systemic lupus erythematosus (SLE) patients treated with telitacicept or belimumab is feasible, with predictors including higher baseline lymphocyte counts, higher serum albumin levels, and telitacicept use, while hematological involvement is associated with a lower likelihood of early LLDAS achievement; these findings highlight the importance of these factors in identifying patients who are likely to benefit from treatment.

L’Estrange-Stranieri et al. reviewed the phosphorylation and protein-binding targets of Lyn, a Src family protein tyrosine kinase, plays a critical role in SLE by influencing B lymphocyte and myeloid cell signaling; research over the past 30 years has revealed that Lyn’s function is affected by both gain-of-function mutations and altered signaling pathways, leading to severe autoinflammation, with studies showing mixed results that may reflect disease heterogeneity and suggest Lyn’s potential role in patient stratification; this review covers Lyn’s phosphorylation and protein-binding targets, its structural domains involved in function, and recent updates on Lyn’s role in SLE.

We hope this Research Topic will help readers address critical questions about cytokine signaling pathways in SLE, advancing our understanding and enabling us to serve patients more effectively.

